# Phylogenomics and Divergence Dating of Fungus-Farming Ants (Hymenoptera: Formicidae) of the Genera *Sericomyrmex* and *Apterostigma*

**DOI:** 10.1371/journal.pone.0151059

**Published:** 2016-07-28

**Authors:** Ana Ješovnik, Vanessa L. González, Ted R. Schultz

**Affiliations:** 1 Entomology Department, National Museum of Natural History, Smithsonian Institution, Washington, District of Columbia, United States of America; 2 Maryland Center for Systematic Entomology, Department of Entomology, University of Maryland, College Park, Maryland, United States of America; 3 Global Genome Initiative, National Museum of Natural History, Smithsonian Institution, Washington, District of Columbia, United States of America; University of Wisconsin-Madison, UNITED STATES

## Abstract

Fungus-farming ("attine") ants are model systems for studies of symbiosis, coevolution, and advanced eusociality. A New World clade of nearly 300 species in 15 genera, all attine ants cultivate fungal symbionts for food. In order to better understand the evolution of ant agriculture, we sequenced, assembled, and analyzed transcriptomes of four different attine ant species in two genera: three species in the higher-attine genus *Sericomyrmex* and a single lower-attine ant species, *Apterostigma megacephala*, representing the first genomic data for either genus. These data were combined with published genomes of nine other ant species and the honey bee *Apis mellifera* for phylogenomic and divergence-dating analyses. The resulting phylogeny confirms relationships inferred in previous studies of fungus-farming ants. Divergence-dating analyses recovered slightly older dates than most prior analyses, estimating that attine ants originated 53.6–66.7 million of years ago, and recovered a very long branch subtending a very recent, rapid radiation of the genus *Sericomyrmex*. This result is further confirmed by a separate analysis of the three *Sericomyrmex* species, which reveals that 92.71% of orthologs have 99% - 100% pairwise-identical nucleotide sequences. We searched the transcriptomes for genes of interest, most importantly *argininosuccinate synthase* and *argininosuccinate lyase*, which are functional in other ants but which are known to have been lost in seven previously studied attine ant species. Loss of the ability to produce the amino acid arginine has been hypothesized to contribute to the obligate dependence of attine ants upon their cultivated fungi, but the point in fungus-farming ant evolution at which these losses occurred has remained unknown. We did not find these genes in any of the sequenced transcriptomes. Although expected for *Sericomyrmex* species, the absence of arginine anabolic genes in the lower-attine ant *Apterostigma megacephala* strongly suggests that the loss coincided with the origin of attine ants.

## Introduction

Fungus-farming (hereafter “attine”) ants are a monophyletic group in which all known species grow fungus for food. The most conspicuous attine ants, the leaf-cutting genera *Atta* and *Acromyrmex*, are dominant herbivores in Neotropical ecosystems [1] and have become model organisms for studies of symbiosis, higher eusociality, and coevolution [2,3]. The attine ants have been divided into two informal groups: the lower and the higher attine ants, the former paraphyletic with respect to the latter. Higher attine ants include, in addition to the leaf-cutters, the non-leaf-cutting genera *Trachymyrmex* and *Sericomyrmex*. All of the higher attine ants grow highly derived, obligately symbiotic fungal cultivars, in contrast to the lower attine ants, which grow facultatively symbiotic cultivars capable of living outside of the symbiosis [2].

The higher attine ant genus *Sericomyrmex* contains 19 nominal species and 3 nominal subspecies and has a broad Neotropical distribution [4,5]. *Sericomyrmex* ants are commonly collected in leaf-litter samples in biodiversity studies in South and Central America, but are very hard to identify to the species level. Species are morphologically very similar and within-nest variation is substantial, confounding easy recognition of species boundaries [6]. In addition, preliminary multiple-gene studies have shown surprisingly low molecular divergence (Ješovnik, *unpublished*), which makes this genus a compelling group in which to study speciation. The phylogenetic position of *Sericomyrmex* within the higher attine ant clade, and its similarities with *Atta* leaf-cutter ants [7], makes understanding the evolutionary history and biology of *Sericomyrmex* species important for reconstructing the origin and evolution of higher attine agriculture and for explaining the ecological success of the leaf-cutting genera *Atta* and *Acromyrmex*.

The lower attine ant genus *Apterostigma*, with 45 described species [8], is remarkable for its symbiotic plasticity. Like all other genera of lower attine ants, one clade of *Apterostigma* species grows fungi in the tribe *Leucocoprineae*, whereas, unlike any other lower or higher attine ant, another clade of *Apterostigma* species cultivates coral fungi in the distantly related family *Pterulaceae* [2]. Most remarkably, a recent study revealed the only known case of a lower attine ant cultivating a higher attine fungus: *A*. *megacephala* grows *Leucoagaricus gongylophorus*, the most highly derived and recently evolved leucocoprineaceous fungal species, an obligate symbiont otherwise known to be cultivated only by leaf-cutting ants [9].

Attine ant genomic studies [10–12] have significantly advanced our understanding of the evolution of fungus-farming in ants. The goal of this study was to sequence, *de novo* assemble, and characterize transcriptomes for species in the genera *Sericomyrmex* and *Apterostigma*, in order to better understand the evolution of fungus-farming behavior and species boundaries within the genus *Sericomyrmex*. Here we report the first genomic data for both genera. For *Sericomyrmex* we sequenced the transcriptomes of three different morphospecies. These three morphospecies were chosen to be the most morphologically and molecularly diverged of the samples assembled for a taxonomic revision of the genus that were properly preserved for RNA extraction. We combined the data produced in this study with published ant and honey bee genomes [10–16] in order to confirm the phylogenetic position of the genus *Sericomyrmex* and to infer divergence dates for *Sericomyrmex*. The transcriptome of *A*. *megacephala* is the first genomic data generated for any species in the Paleoattini, one of the two sister clades produced by the basal-most divergence in the fungus-farming ant phylogeny. The other sister clade, the Neoattini, includes the higher attines and all previously sequenced species, so genetic data for a paleoattine species significantly improves our ability to date the loss of arginine biosynthesis.

## Materials and Methods

### Sample preparation and sequencing

No animal ethics approvals are required to conduct research on ants. Research in Brazil was covered by Brazilian Council of Research and Scientific Development permit Processo CNPq 001884/2012-3 and Instituto Chico Mendes de Conservação da Biodiversidade (ICMBio) collecting permit 14789–6. Research in Peru was covered by Ministerio de Agricultura Instituto Nacional de Recursos Naturales INRENA Autorización No. 034-2004-INRENA-IFFS-DCB; Modificación a la Autorización No. 034-2004-INRENA-IFFS-DCB; Ministerio de Agricultura Instituto Nacional de Recursos Naturales INRENA Autorización No. 12 C/C-2004-INRENA-IANP; Carta No. 553-2014-MINAGRI-DGFFS/DGEFFS; Ministerio de Agricultura Instituto Nacional de Recursos Naturales INRENA Autorización No. 088-2005-INRENA-IFFS-DCB; CARTA No. 0217-2012-SERNANP-JRNTAMB; Resolución del Jefe de la Reserva Nacional Tambopata No. 020-2012-SERNANP-JEF; Permiso para Fauna y Flora Silvestre No. 004905-AG-INRENA; Permiso para Fauna y Flora Silvestre No. 006771-AG-INRENA; and Permiso para Fauna y Flora Silvestre No. 009154-AG-DGFFS.

Specimens of three *Sericomyrmex* species were collected from live colonies in the field and preserved in RNAlater. Specimens of *A*. *megacephala* were collected from a live laboratory nest and also preserved in RNAlater. Voucher specimens for each of the species sequenced are deposited in the Department of Entomology, National Museum of Natural History, Washington, DC, USA (Table 1), as recorded in the NMNH K-EMu database (http://collections.nmnh.si.edu/search/ento/). In order to ensure sufficient quantities of RNA, we included ten workers per sample for each *Sericomyrmex* species and five workers for the larger species, *A*. *megacephala*, crushing them with sterilized wooden sticks to enable the RNAlater to rapidly penetrate the integument. All specimens were stored at -20°C until RNA extraction. We extracted RNA using the Promega SV Total RNA Isolation System, following the standard protocol. The Institute for Bioscience and Biotechnology Research (IBBR) at the University of Maryland prepared libraries using the standard Illumina TruSeq RNA Sample Preparation protocol (all libraries were normalized and redundant rRNA was removed), and performed 100bp pair-end sequencing on Illumina HiSeq.

**Table 1:** Specimen data.

	*Apterostigma megacephala*	*Sericomyrmex* cf. *mayri*	*Sericomyrmex* cf. *parvulus*	*Sericomyrmex* cf. *luederwaldti*
Collection code	JSC110911-16	AJ120726-03	AJ120728-10	AJ121019-03
Collection date	11 September 2011	26 July 2012	28 July 2012	19 October 2012
Preservation method	RNAlater	RNAlater	RNAlater	RNAlater
Country	Brazil	Peru	Peru	Brazil
Locality	Para, Carajas	Madre de Dios, Tambopata NP	Madre de Dios, Tambopata NP	Minas Gerais, Panga Field Station
Habitat	Disturbed rain forest	Primary rain forest	Primary rain forest	Cerrado *sensu stricto*
Coordinates	6.06321 S, 50.05776 W	12.81867 S, 69.36364 W	12.85001 S, 69.37215 W	19.18314 S, 48.40141 W
Elevation	676 m	224 m	196 m	813 m
Voucher Specimen	USNMENT01124335	USNMENT01124333	USNMENT01124334	USNMENT01124336

### Data cleaning and assembly

**Fig 1:**
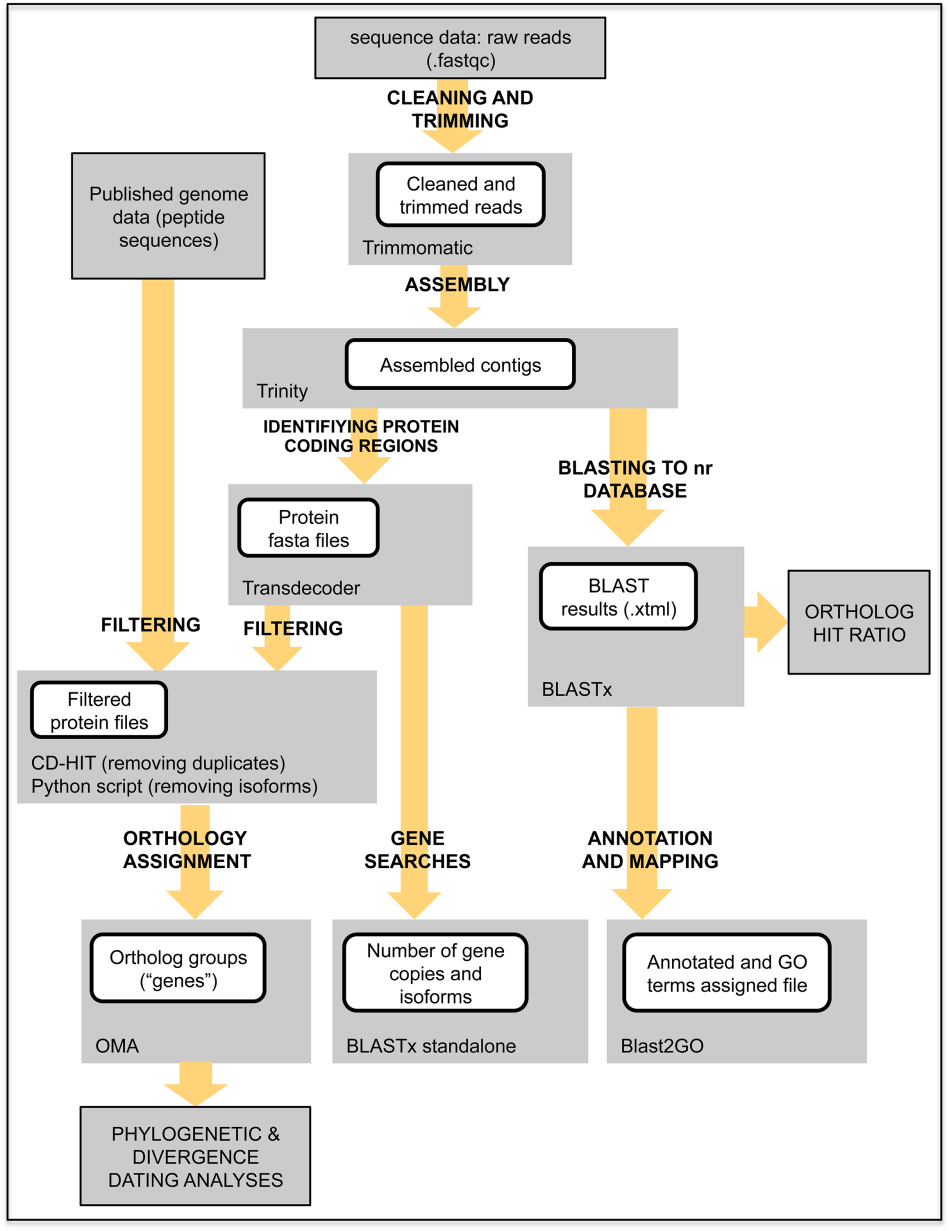
Workflow summary.

We concatenated the raw data and performed a quality check with FastQC [17] before and after trimming (Fig 1). Raw reads were cleaned and trimmed with Trimmomatic [18] and the resultant cleaned reads were assembled with Trinity [19,20] using the default parameters. Basic assembly statistics (number of transcripts, average transcript length, and N50) were obtained from Trinity. We processed the resulting assemblies in TransDecoder [21] to identify candidate coding regions within the transcripts. Predicted peptides were then filtered with CD-HIT-EST (98% global similarity) [22] to remove redundant sequences. After removal of duplicates, peptides were further filtered in order to select only one peptide per putative transcript by choosing the longest ORF (Open Reading Frame) per Trinity subcomponent with a custom-made Python script [23]. This step ensured that we had removed variation in the coding regions of our assemblies due to alternative splicing, closely related paralogs, and allelic diversity. Peptide sequences for the seven sampled ant genomes were filtered at 98% similarity in CD-HIT-EST. To calculate coverage we multiplied the number of raw reads with the expected length (100 bp), and divided that by total number of bases in the assembly [24].

### Orthology Assignment and Alignment

We identified orthologs using OMA stand-alone v.0.99w software [25,26] with default settings on 100 cores on the Smithsonian Lattice high-performance computing cluster (Linux-based with AMD processors). We constructed an amino acid supermatrix by concatenating the set of OMA groups containing all taxa (1,317). Each putative ortholog group (from now on “gene”) was aligned individually using MAFFT [27]. Aligned genes were then trimmed with Gblocks [28] to cull regions of dubious alignment.

We separately ran an orthology assignment in OMA for a data subset containing only the *Sericomyrmex* transcriptomes. For this we used TransDecoder to identify the nucleotide sequences for the candidate protein-coding regions and performed the same filtering as described above with the larger dataset on the resultant nucleotide sequence data. Individual gene alignments were trimmed with Gblocks [28] and analyzed in Geneious v.8 [29].

### Phylogenetic analyses

Maximum-likelihood inference was conducted with PhyML-PCMA [30,31]. We selected 10 PCs (principal components) in the PhyML-PCMA analyses and used empirical amino-acid frequencies. PhyML-PCMA estimates a model through the use of a principal component (PC) analysis; in this case using 10 PCs. Bootstraps were calculated in PhyML-PCMA for 100 replicates. Concomitantly, tree searches were conducted in PhyloBayes MPI 1.4e [32] using the site-heterogeneous CAT + GTR model of evolution. Three independent chains were run for 1295–1445 cycles, and the initial cycles discarded as *burn-in* were determined for each analyses using the “*tracecomp*” executable, with convergence assessed using the maximum bipartition discrepancies across chains (maxdiff < 0.1).

### Divergence dating

Divergence dates were estimated using the Bayesian relaxed molecular clock approach as implemented in PhyloBayes v.3.3f [32] applying an auto-correlated model of clock relaxation [33,34]. Three calibration constraints (S1 Table), based on a recent study of myrmicine ants [35], were used with soft bounds [36] under a birth-death prior in PhyloBayes. PhyloBayes was run for 32,664 cycles, sampling posterior rates and dates every 5 cycles. The initial 5000 cycles were discarded as burn-in. We excluded the outgroup taxon *Apis mellifera* from final analyses because we considered it to be an inappropriate outgroup for the purposes of divergence dating. All other taxa are members of a single subfamily, the Myrmicinae, which occupies a highly derived position within the ant family Formicidae, whereas *A*. *mellifera*, the honey bee, is a highly derived member of a highly derived family within the superfamily Apoidea. The most recent common ancestor of the lineages containing the Apoidea and the Formicidae dates to at least 140 mya, whereas the ancestor of the Myrmicinae, the focus of our dating analyses, dates to ~99 mya [35,37,38]

### Annotation and Ortholog Hit Ratio

We used BLASTx [39] (cut-off E-value 1e^-5^) to compare the unfiltered, assembled transcripts (Trinity output) against the non-redundant (nr) protein database of NCBI. Resulting xml files were used as input for CLC Workbench (CLC Inc., Aarhus, Denmark). We performed functional annotation and mapping to GO terms, using Blast2GO with default settings, in order to summarize functional categories of the genes, annotate our data set, and determine the quality of our transcriptomes. We also used the resultant xml output to run Orthology Hit Ratio calculations following previously described methods and scripts [40]. This analysis estimates the degree to which a transcriptome is fully sequenced and assembled by comparing the length of the contigs that recovered BLAST hits with the length of their top BLAST hits [41,42].

### Gene search

Based on previous studies of attine ant genomes and ant transcriptomes [10–12,43] we considered the following genes or gene families of particular interest: genes in the arginine metabolism pathway (*arginase*, *nitric oxide synthase*, *argininosuccinate synthase*, and *argininosuccinate lyase*), detoxification genes (*cytochrome P450 monooxygenase*), hexamerins (*hex 70a*, *hex 70b*, *hex 70c* and *hex 110*), desaturase, RYamide, and chitinases. The nucleotide or protein sequence for each of these genes was downloaded from NCBI GenBank or, in the case of the desaturase gene, obtained from the supplemental material of a previous study [43]. We used sequences from the closely related species *Atta cephalotes* and *Acromyrmex echinatior* for searches for *arginase*, *nitric oxide synthase*, and *cytochrome P450*. For chitinase searches we assembled protein and nucleotide sequences that were used in similar analyses in a study of the *Atta cephalotes* genome [10], for a total of ten different chitinase-like proteins from *Acromyrmex echinatior*, *Nasonia vitripennis*, and *A*. *mellifera* (S4 Table). For *argininosuccinate synthase* and *argininosuccinate lyase*, which are lost in leaf-cutter ants, we used copies from the closely related myrmicine ants *Solenopsis invicta*, *Pogonomyrmex barbatus*, and *Wasmannia auropunctata*. For hexamerin searches we used *Apis mellifera* sequences in order to repeat the methods of previous studies [10] and because some of the attine ant hexamerin sequences were unavailable. For searches for ryamide, which is absent in ants, we used a *Drosphila melanogaster* sequence. Full sequences of all genes used in our analyses, with GenBank accession numbers, can be found in S1 Text. We used BLAST+ standalone [44] to manually create a BLAST database for each of the transcriptomes separately (using unfiltered, assembled transcripts) and searched the created databases for each of the query sequences with the cut-off E-value 1e^-5^. Returned hits were then BLASTed against NCBI to confirm that they had returned the same protein.

## Results and Discussion

We sequenced the transcriptomes of the lower attine ant *Apterostigma megacephala* and three morphospecies of the higher attine ant genus *Sericomyrmex* (Fig 1, Table 1). The total number of raw sequences was 329.6 Mb, varying from 72 to more than 91 million reads per sample. After trimming and cleaning, between 92.2% and 93.8% of the raw reads were used for assembly. The number of assembled contigs varied from 71,391 for *S*. cf. *luederwaldti* to 95,242 for *A*. *megacephala*. The N50, contig length, and other statistics for each of the sequenced taxa can be found in Table 2. The number of raw reads and assembled contigs were highest for *A*. *megacephala*, which can be attributed to the high quality of preservation of the extracted RNA for this sample.

**Table 2:** Transcriptome sequencing, assembly, and analysis statistics. Sources of statistics: 1: FastQC, 2: Trimmomatic, 3: Trinitystats.pl, 4: Geneious, 5: Transdecoder, 6: Blast2GO, 7: calculated from other statistics, 8: Ortholog Hit Ratio.

	*Apterostigma megacephala*	*Sericomyrmex* cf. *mayri*	*Sericomyrmex* cf. *parvulus*	*Sericomyrmex* cf. *luederwaldti*	Stats. source
Number of raw reads after sequencing	91 666 468 (91,6 Mb)	85 655 146 (85, 6 Mb)	72 383 100 (72,3 Mb)	80 111 038 (80,1 Mb)	1
Percentage of reads after trimming (= percent of reads used for assembly)	93.87%	92.29%	92.85%	92.56%	2
Number of transcripts (contigs) after assembly	95242	65983	83935	71391	3
Assembled contigs length: min-max (average, st dev)	201–30835 (1670.9, 2177.2)	201–20168 (1358.4, 1551.6)	201–28459 (1601.9, 1894.6)	201–28605 (1405.2, 1665.9)	4
N50	3466	2755	3168	2739	3
Number of potential protein-coding regions	38113	33585	40801	37563	5
Number of contigs without BLAST hits (%)	44986 (49.11%)	31597 (48.28%)	38205 (46.60%)	31133 (44.11%)	6 (7)
Number of contigs with BLAST results (%)	17558 (19.16%)	10659 (16.28%)	15165 (18.49%)	14277 (20.22%)	6 (7)
Number of contigs with mapping results (%)	3212 (3.5%)	1630 (2.49%)	1796 (2.19%)	1239 (1.75%)	6 (7)
Number of annotated contigs (%)	25843 (28.21%)	21555 (32.93%)	26808 (32.70%)	23925 (33.90%)	6 (7)
Total number of sequences annotated with BP (Biological Processes)	50366	46495	52420	47920	6
Total number of sequences annotated with MF (Molecular Function)	32999	27495	34303	30926	6
Total number of sequences annotated with CC (Cellular Component)	20098	18641	21954	18944	6
Species with highest number of BLAST top-hits	*Acromyrmex echinatior*	*Acromyrmex echinatior*	*Acromyrmex echinatior*	*Acromyrmex echinatior*	6
Species with highest number of BLAST hits	*Cerapachys biroi*	*Acromyrmex echinatior*	*Acromyrmex echinatior*	*Acromyrmex echinatior*	6
Number of contigs with OHR >0.5	72.24%	64.60%	69.50%	63.70%	8
Number of contigs with OHR >0.8	55.15%	49.70%	53.50%	47.80%	8
COVERAGE	57.6	169.7	53.8	79.8	7
NCBI SRA BIoSample Accession Number	SAMN04166284	SAMN04166285	SAMN04166286	SAMN04166287	

**Fig 2:**
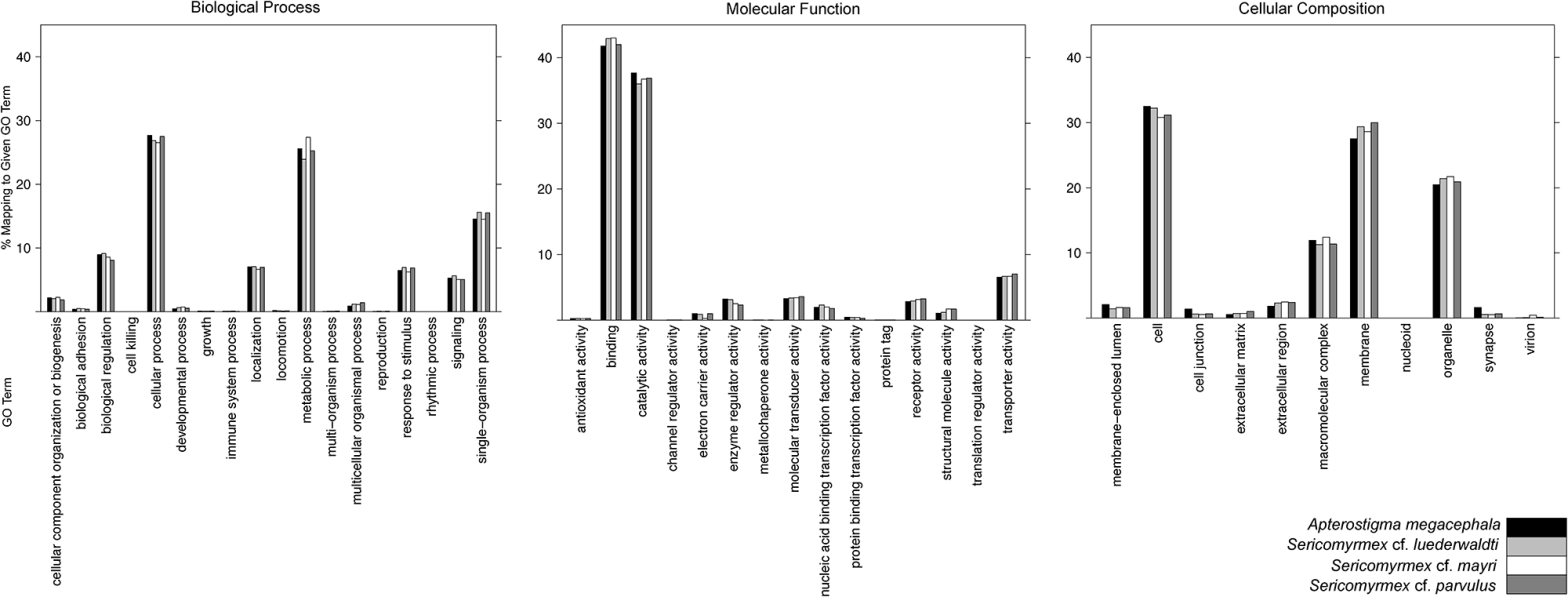
Gene Ontology (GO) distributions by level, by species.

**Fig 3:**
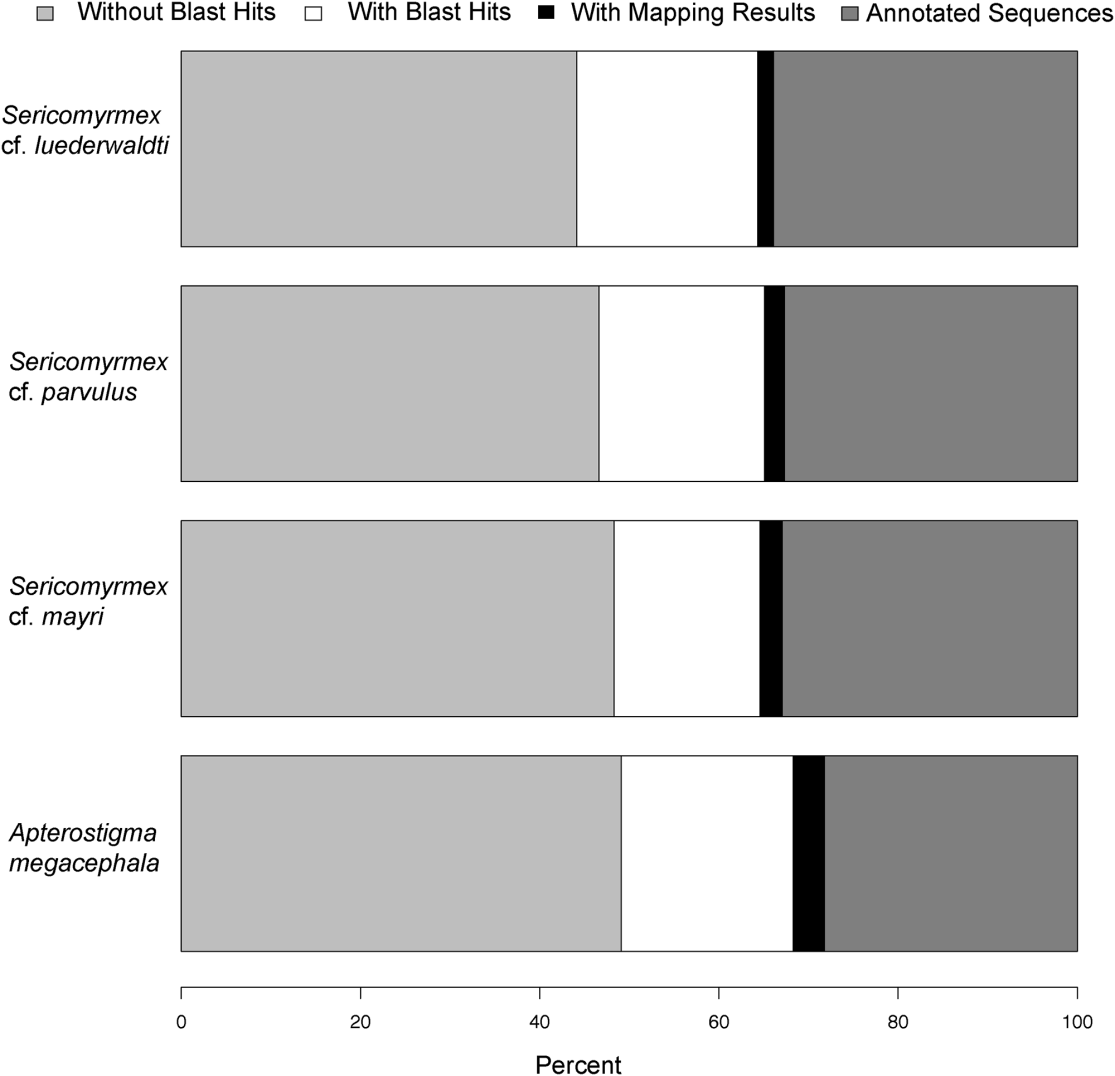
Functional annotation. Percentages of BLAST-hits, annotated sequences, mapped sequences, and no-BLAST hits for each species.

Coverage for the transcriptomes varied from 53.8 to 169.7 reads per base (Table 2). Ortholog Hit Ratios (OHR) were calculated in order to estimate transcriptome assembly quality and completeness [42]. An OHR value close to 1 suggests complete transcript assembly, with a value of 1 being an identical match. On average, our transcriptomes had high OHR values, with 47.7 to 53.5% of total contigs having an OHR greater than 0.8, and 63.7 to 69.5% of contigs having an OHR higher than 0.5 (Table 2, S1 Fig). A number of contigs recovered values over 1, which is not uncommon [41], and represents possible sequence expansions. Additionally, we calculated annotation metrics to estimate transcriptome quality (e.g., number of contigs that recovers at least one BLAST hit). Based on these metrics (Figs 2 and 3, Table 2, S2 and S3 Tables), all four transcriptomes are of high quality with values that are either within or slightly higher than those reported in similar studies [24,40,43].

### Orthology assignment

We used OMA standalone software [26] to place predicted genes into orthologous groups. OMA’s advantage over standard bidirectional best-hit approaches is that OMA’s algorithm uses evolutionary distances instead of scores, considers distance inference uncertainty, includes many-to-many orthologous relations, and accounts for differential gene losses [26,45]. The number of orthologs obtained for the complete dataset (ten ingroup and three outgroup taxa) was 1,317 and the number of phylogenetically informative genes (n>4) was 11,447. The final concatenated matrix contains a total of 649,095 amino-acid sites present in all taxa.

### Functional annotation

The percentage of reads that had at least one BLAST hit varied between 50% and 55.8% of total transcripts (Table 2), which is slightly higher than in similar transcriptome studies [40,43]. Final number of annotated sequences varied between 21,555 and 26,808 sequences. Our data were annotated with a wide range of functional categories represented on all levels of the Gene Ontology database and were comparable to other similar studies, with no biases toward any particular category (Fig 2, Table 2). For all four transcriptomes the top BLAST hit species was recovered as *Acromyrmex echinatior*, which is a higher-attine leaf-cutting species, a very close relative of *Sericomyrmex*, and a close relative of *Apterostigma*. This was also the species with the largest number of total BLAST hits for the three *Sericomyrmex* species. For *A*. *megacephala* the top BLAST hit species was the non fungus-farming ant *Cerapachys biroi*.

### Phylogeny and dating

**Fig 4:**
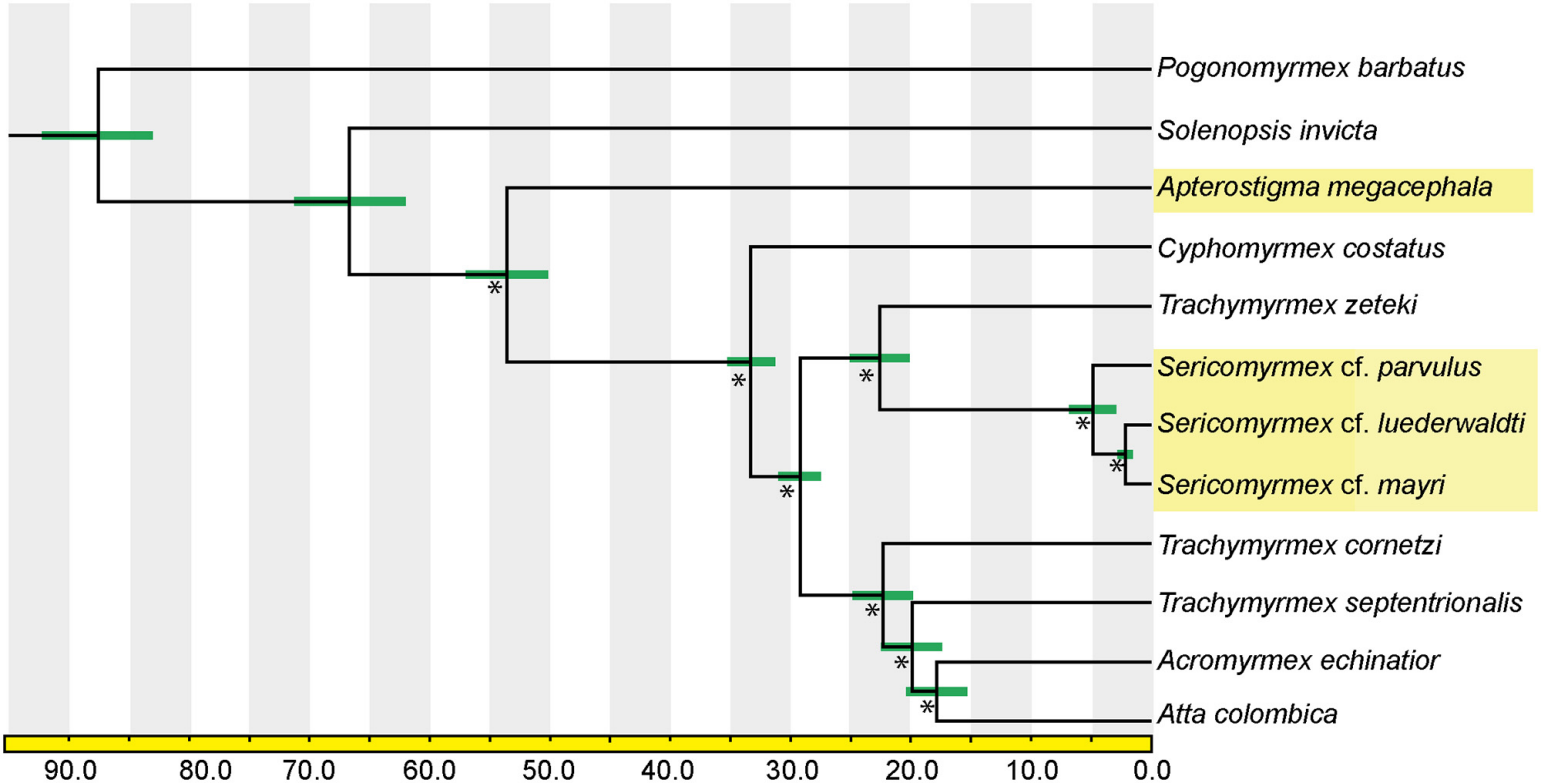
Time-calibrated phylogeny of attine ants. Highlighted taxa are novel transcriptomes obtained for this study. This topology was recovered in both ML and Bayesian analysis. Asterisks (*) at nodes indicate bootstrap values of 100 and posterior probabilities of 1.0. Green error bars at nodes indicate minimum and maximum age estimates; the time scale at the bottom is in million of years.

Results from phylogenetic analyses of this dataset (Fig 4) are congruent with existing phylogenies, including a monophyletic *Sericomyrmex* clade that is the sister of *Trachymyrmex zeteki*, and with *A*. *megacephala* as the sister taxon to all other attine ant taxa included in this study. Considering the large number of characters in this dataset, the branches subtending the three *Sericomyrmex* species are reconstructed as remarkably short in both analyses. This result indicates a very low genetic divergence between *Sericomyrmex* species. To investigate this further we ran a separate orthology search including only the three *Sericomyrmex* species, which revealed 4,217 orthologous genes present in all three samples, varying in length from 303 to 10,530 bp. Of those 4,217 loci, 92.71% (3,910 sequences) are 99% to 100% pairwise identical, as defined by Geneious [29]. Of the remaining orthologs, 290 are between 90% and 99% pairwise identical. Combining these results, 99.59% of all orthologs shared by these three *Sericomyrmex* morphospecies are at least 90% pairwise identical. This result is unexpected given that we selected the most molecularly and morphologically divergent *Sericomyrmex* samples available for RNA extraction. Based on morphology and multiple gene sequences, of the samples chosen for this study *S*. cf. *luederwaldti* and *S*. cf. *mayri* were considered *a priori* to be more similar, so the high similarity of their transcriptome sequences is less surprising. However, *S*. cf. *parvulus* is morphologically distinctly different from the other two species and is considered to be a member of one of the most basally diverging lineages of *Sericomyrmex* species (Ješovnik, *unpublished*). Even though phylogenetic analyses of the transcriptome data agreed with prior analyses regarding these relationships, i.e., that *S*. cf. *luederwaldti* and *S*. cf. *mayri* are more closely related to each other than either is to *S*. cf. *parvulus*, the degree of divergence separating *S*. cf. *parvulus* from those two species is much lower than expected based on divergences separating similarly related taxa, e.g., some leaf-cutter species. In general, the transcriptomes indicate that even morphologically divergent *Sericomyrmex* species are separated by remarkably low genetic distances, which means that accurately recovering species boundaries may require more variable data from non-coding regions. Importantly, this pattern, also supported by our divergence-dating analyses (see next paragraph) indicates that *Sericomyrmex* has rapidly radiated into multiple, morphologically distinguishable species that occupy a broad geographic distribution [4,46] with only a small degree of accompanying genetic divergence, in contrast to most other comparable ant groups, including its sister taxon, the *Trachymyrmex iheringi* species group. This conclusion must obviously be investigated further, given that transcriptomes from only three *Sericomyrmex* species were included in this study. It will be particularly important to test whether this rapid radiation may have been driven by coevolution with a specialized clade of higher attine fungi, as has been suggested for *Atta* [2,47], especially since recent research suggests the possibility of high symbiont fidelity between *Sericomyrmex* species and a single species of fungal cultivar [48]. Another potentially important factor in this radiation could be major changes in genetic architecture such as chromosome duplications and rearrangements, because data from two species indicate that, compared to most other attine ants, *Sericomyrmex* has an unusually high number of chromosomes and an unusually large genome [49,50].

The divergence dating analysis, which included ant species only (the outgroup *A*. *mellifera* was excluded in the dating analysis), recovered the stem-group age (i.e., the earliest possible origin) of fungus-farming ants as 66.7 million years ago (mya) and the crown-group age (i.e., the latest possible origin) as 53.6 mya. The crown-group age estimate is consistent with previous estimates of 50–60 mya [2,9,35,50,51], whereas the stem-group age estimate is slightly younger than a stem-group estimate from a recent study (74.6 mya) of a large genomic data set [12]. However, these results are in some cases imprecise and are not directly comparable because neither included taxa from the closest non-fungus-farming relatives (the sister group) of the attine ants [35] and only this study included representatives of both clades resulting from the basal-most divergence at the origin of the fungus-farming ants. Our age estimate (33.3–53.6 mya) of this divergence between the Paleoattini (including the genera *Myrmicocrypta*, *Apterostigma*, and *Mycocepurus*) and the Neoattini (including all other attine ant genera) should be interpreted with caution because our taxon sampling is arguably inadequate for the task. Our taxon sampling for the higher attine ants is better and represents the first genomic data set that includes *Sericomyrmex*. We estimate the crown-group age of the higher attine ants to be 29.2 (31.3–27.4) mya; the crown-group age of the *Sericomyrmex*+*Trachymyrmex zeteki* clade (i.e., the *Trachymyrmex iheringi* group) as 22.6 (25.3–20.4) mya; and the crown-group age of the leaf-cutting ants (genera *Atta* and *Acromyrmex*) to be 17.9 (20.4–15.6) mya (Table 3). For comparison, Schultz and Brady [2] recovered ~16 mya, ~10.5, and ~8 mya mya for those same nodes, respectively, and Nygaard and colleagues estimate ~17 mya for the leaf-cutter origin [12]. Our dating analyses, based on transcriptome data, are most likely overestimating divergence times due to the very small taxon sample. This possible overestimation notwithstanding, the crown-group age of *Sericomyrmex* is reconstructed as very recent, estimated at 4.8 mya for the age of the ancestor of all extant species. For comparison, the stem- and crown-group ages of the clade containing the attine host/parasite species pair *Mycocepurus goeldii* and *M*. *castrator* are estimated to be 2.04 and 3.31 million years, respectively [52], and crown-group divergences between sister species pairs in the *Cyphomyrmex wheeleri* group range from 5.3 to 7.0 mya [3]. The branch separating *Sericomyrmex* from the most recent common ancestor it shares with the *Trachymyrmex iheringi* group (represented here by *T*. *zeteki*) is very long (Fig 4), which might be explained by taxon undersampling, especially if there are more extant *Sericomyrmex* species that could split that long branch if they had been included. We consider this unlikely, however, because the three sampled *Sericomyrmex* species were chosen to represent the full range of diversity in the genus both morphologically and molecularly and because other phylogenomic studies currently in preparation, employing much denser taxon sampling, corroborate that the genus *Sericomyrmex* is the sister group of the entire *T*. *iheringi* clade, i.e., that the branch subtending *Sericomyrmex* is unoccupied by other *Trachymyrmex* species.

**Table 3:** Crown-group and stem-group age estimates. In units of million years ago, with standard error given in parentheses. An asterisk (*) indicates a posterior probability of 1.

Clade	This study	Schultz & Brady 2008	Nygaard et al. 2016
All attine ants			
Crown	53.6 (56.7–49.2)*	**50 (56–44)**	
Stem	66.7 (70.7–61.6)*	**51(57–45)**	**74.63 (84.28–65.24)**
Neoattini (all attine ants except *Apterostigma)*			
Crown	33.3 (35.1–31.3)*		
Stem	53.6 (56.9–49.2)*		
Higher attine ants *(Trachymyrmex*, *Sericomyrmex*, *Atta*, *Acromyrmex)*			
Crown	29.2 (31.3–27.4)*	**16 (19–13)**	**22.87 (17.17, 28.57)**
Stem	33.3 (35.1–31.3)*	**20 (24–17)**	**26.60 (19.60, 33.80)**
*Trachymyrmex zeteki +* genus *Sericomyrmex*			
Crown	22.6 (25.3–20.4)*	**10.58**	
Stem	29.2 (31.3–27.4)*	**16**	
Genus *Sericomyrmex*			
Crown	4.9 (7.1–3.4) *		
Stem	22.6 (25.3–20.4)*		
*Sericomyrmex cf*. *mayri + Sericomyrmex cf*. *luederwaldti*			
Crown	2.1 (3.4–1.4)*		
Stem	4.9 (7.1–3.4) *		
*Trachymyrmex cornetzi*, *T*. *septentrionalis +* leafcutter ants			
Crown	22.3 (25.1–20.1)*		**19.18 (23.57–14.69)**
Stem	29.2 (31.2–27.4) *		**22.87 (17.17, 28.57)**
*Trachymyrmex septentrionalis +* leafcutter ants			
Crown	19.9 (22.5–17.7)*		**17.77(21.72–13.70)**
Stem	22.3 (25.1–20.1)*		**19.18 (23.57–14.69)**
Leafcutter ants: *Atta colombica + Acromyrmex echinatior*			
Crown	17.9 (20.4–15.6)*	**8 (10–6)**	**7.05 (8.44–5.60)**
Stem	19.9 (22.5–17.7)*	**9 (7–11)**	**16.21 (19.74–12.57)**

### Gene family searches

In order to further investigate the sequenced transcriptomes, we chose several genes of interest based on previous ant genome and ant transcriptome studies [10,11,43] and performed separate searches for those genes. In general, the similarity of the results of our gene BLAST searches to those of other attine ant genomic studies serves as a confirmation of the completeness of our transcriptomes.

#### Arginine metabolic pathway genes

The first genomic studies of the higher-attine leaf-cutting ant genera *Atta* and *Acromyrmex* demonstrated losses of two genes in the arginine anabolic pathway, whereas all other ants for which data are available, including species in the same subfamily (Myrmicinae) as the attine ants, have functional copies of those genes [10,11]. It was suggested that the leaf-cutting ants, or even all higher attine ants, had become obligately dependent on their fungi for arginine. Such a metabolic division of labor is perhaps not surprising in the highly derived higher attine ants, given that their fungi are also obligate symbionts, and similar examples are known from other mutually obligate symbioses [53–55]. However, a more recent study, which included one lower attine ant species that is relatively closely related to the higher attines, indicates that those two genes, *argininosuccinate synthase* and *argininosuccinate lyase*, were lost prior to the origin of the higher attines [12], begging the question of at what point in attine evolution the ants became obligately dependent on their fungal cultivars for the amino acid arginine.

Our BLAST searches for *argininosuccinate synthase* and *argininosuccinate lyase* in the three *Sericomyrmex* species did not retrieve any hits, which, because *Sericomyrmex* species are higher attines, was to be expected. However, BLAST searches also failed to find these genes in *A*. *megacephala*. *A*. *megacephala* is a member of the Paleoattini, one of two sister clades formed by the basal-most divergence in the fungus-farming ant phylogeny. The other sister clade, the Neoattini, contains *Sericomyrmex* and all previously studied attine ant species, so the absence of these two genes in a paleoattine indicates that the dependence of attine ants on their fungal cultivars for arginine arose in the common ancestor of all fungus-farming ants. This result is unlikely to be due to inadequate transcriptome data for two reasons. First, our Ortholog Hit Ratios are high, and, second, searches for two other enzymes in the arginine metabolic pathway, the catabolic genes *arginase* and *nitric oxide synthase*, produced hits in all of our transcriptomes (Table 4). Functional copies of these two genes are likewise present in the genomes of *Atta* and *Acromyrmex* [10,11].

**Table 4:** Gene searches. Number of gene copies and isoforms per species.

	*Apterostigma megacephala*		*Sericomyrmex* cf. *mayri*		*Sericomyrmex* cf. *parvulus*		*Sericomyrmex* cf. *luederwaldti*	
	# of copies	# of isoforms	# of copies	# of isoforms	# of copies	# of isoforms	# of copies	# of isoforms
Hexamerins	**3**	**0**	**4**	**0**	**4**	**0**	**2**	**0**
Desaturase	**5**	**9**	**8**	**11**	**8**	**12**	**8**	**15**
CytP450	**31**	**106**	**42**	**118**	**38**	**117**	**38**	**97**
Arginase	**4**	**0**	**3**	**0**	**4**	**0**	**9**	**0**
Nitric Oxide Synthase	**7**	**0**	**26**	**0**	**10**	**0**	**10**	**0**
Chitinases	**9**	**0**	**10**	**0**	**10**	**0**	**10**	**0**

#### Hexamerins

Hexamerins are another protein family that has been associated with the specialized diet of attine ants [10]. Four copies of hexamerins are commonly found in insects, *hex 70a*, *hex 70b*, *hex 70c*, and *hex 110*, serving as storage proteins during development as well as in the adult stage [56]. In the genome study of the leaf-cutting ant *Atta cephalotes*, the gene *hex 70c* was not found [10]. BLAST searches with *Apis mellifera* hexamerins recovered two to four different hexamerins per ant transcriptome (Table 4), but unfortunately we were not able to distinguish between the different copies. In the transcriptome of *S*. cf. *luederwaldti* our search recovered only two copies.

#### RYamide

We found no gene sequences for the RYamide protein in the *Sericomyrmex* and *Apterostigma* transcriptomes. RYamide proteins are recently discovered and have been found in all insects for which genomes are available [57] except for ants. The functional roles of RYamides are poorly known, but it has been suggested that in ants the absence of RYamide is connected with the differentiation between reproductive and non-reproductive castes [11].

### Desaturases

The desaturase gene family plays an important role in the synthesis of cuticular hydrocarbons (CHC), which are one of the key elements of nestmate recognition in social insects. Using the *desat1* gene of the ant *Formica exsecta* [43] as a query sequence, we found five copies of desaturase in *A*. *megacephala* and eight copies in each of the three *Sericomyrmex* species, all with various numbers of isoforms (Table 4). When the found copies were BLASTed back to the NCBI protein database, the highest similarity was found with delta(Δ)11 desaturase genes of other ants. This finding is comparable to that of a similar study of the *Atta cephalotes* genome, in which six out of eleven identified desaturase-like genes matched Δ11 desaturase [10].

#### Cytochrome P450

Cytochrome P450 (*CytP450*) is a large protein family, members of which are found in enzymatic pathways central to the metabolism of toxic compounds as well as to development and reproduction [58]. We found 31 copies of *CytP450* in *A*. *megacephala*, and between 38 and 42 copies in the three *Sericomyrmex* morphospecies (Table 4). These numbers are surprisingly small. The 54 copies of *CytP450* identified in *Atta cephalotes* is regarded as a reduced number in comparison to other ants (136 in *Camponotus floridanus* and 107 in *Harpegnathos saltator*) [59]. Like fungus-farming ants, both of the two other insects with known low numbers of *CytP450*, the honey bee *Apis mellifera* (with 62 copies) and the body louse *Pediculus humanus* (with 39 copies) [60,61] have specialized diets and it has been suggested that insects with predictable diets may have a reduced need to metabolize toxins.

#### Chitinases

Chitinases, enzymes with chitinolytic activity, play important roles in digestion and moulting in insects. The number of chitinases in Hymenoptera is considered reduced in comparison to that in *Drosophila melanogaster* [10]. In studies of attine ants, however, chitinases have been shown to have experienced positive selection, presumably because of their importance in the digestion of the chitinous cell walls of their fungal cultivars [12]. We found 10 chitinase-like genes, in different copy numbers, in all three *Sericomyrmex* species. In *A*. *megacephala* we found 9 of them, with *CG8460*, chitinase-like protein 1, absent. Interestingly, chitinase gene *K06A9*.*b*, which is found in *A*. *mellifera* and *A*. *cephalotes* but is not known in *D*. *melanogaster*, was found in all of our transcriptomes. It was beyond the scope of this paper to test for positive selection in the chitinase genes, but we found 13–23 copies of *Chitinase 3*, for which positive selection has been detected in other attine ants [12]. Details on copy numbers for each of the chitinases for each of the species can be found in S4 Table.

Our transcriptomes were constructed from worker-caste ants only, and some of the identified genes of interest, including the arginine metabolic pathway and *CytP450* genes, are expressed at higher levels in the larval stage [10,58]. Their absence in our assemblies could therefore be attributed to insufficient sequencing depth due to low levels of expression. This seems unlikely, however, because, as noted above, our Ortholog Hit Ratios are high and we detected the other genes in the arginine metabolic pathway, *arginase* and *nitric oxide synthase*. Rather, our results suggest that the reduction in *CytP450* genes and the losses of *argininosuccinate synthase* and *argininosuccinate lyase* likely occurred at the origin of fungus-farming ants. An alternative hypothesis, at least for the arginine metabolic pathway genes, is that they were lost twice, once in *A*. *megacephala* and once in the ancestor of the higher attine ants. This appears initially plausible because *A*. *megacephala* is the only lower attine known to cultivate a higher-attine fungal cultivar and it is clear that *A*. *megacephala* secondarily acquired its cultivar during the course of its evolution [9]. Hence, if arginine dependence is specifically associated with higher attine fungal cultivars, then it could have occurred in parallel in the higher attine ants and in *A*. *megacephala*. On closer inspection, we consider this hypothesis unlikely because the two arginine anabolic genes are also absent in the lower attine ant *Cyphomyrmex costatus*, which cultivates a lower-attine fungus. Clearly, genomic data from other species of paleoattine and basally diverging neoattine lineages are needed to better understand the history of genomic evolution in the fungus-farming ants.

## Conclusions

Results from our phylogenetic and dating analyses suggest that the genus *Sericomyrmex* has undergone a very recent, rapid diversification, reflected by short branch lengths and recent divergence dates. Most surprisingly, the overall genetic similarity between the three *Sericomyrmex* morphospecies is unexpectedly high. It is our hope that these results will inspire further investigation into the genetic mechanisms underlying rapid radiation with little genetic change in *Sericomyrmex*. In addition to this phenomenon and its implications for speciation, recent research on social parasites and agro-predators makes this genus an exciting model system for studying behavioral ecology, coevolution, and chemical ecology [62–64]. The transcriptomes sequenced in this study could provide the foundation for future research in gene discovery, phylogenomics, population genomics, and conservation genetic studies [65,66]. In our analyses of gene families (arginine metabolic pathway, *Cytochrome P450*, hexamerins, RYamide, desaturase, and chitinase) we failed to find two arginine anabolic enzymes in the paleoattine ant *A*. *megacephala*, suggesting that the loss of these enzymes, confirmed previously only in the clade Neoattini, was likely lost at the origin of the fungus-farming ants. We recovered a surprisingly small number of *CytP450* genes, which are associated with detoxification and unspecialized diets. Additional research is required to confirm these gene losses, since our data do not include larval transcripts, and therefore cannot be interpreted as conclusive. Finally, we hope the transcriptome of *A*. *megacephala* will prove a useful tool for future research on the underlying genetics that makes this species the only lower attine ant known to cultivate a higher attine fungus [9].

## Supporting Information

S1 Fig Ortholog Hit Ratio graphs. Ortholog Hit Ratio values for each of the species sequenced.
(PDF)

S1 Table PhyloBayes dating analysis calibrations. Calibration priors used in PhyloBayes divergence dating analysis. Unit: Million of years ago.
(XLSX)

S2 Table GO distributions by level. GO distributions by level, by species, with GO-ID numbers, the output of BLASTtoGO Annotation analyses.
(XLSX)

S3 Table Enzyme code distribution. Number of sequences annotated with different Enzyme code per species, the output of BLASTtoGO Annotation analyses.
(XLSX)

S4 Table Chitinase genes. Number of chitinase genes copies in transcriptomes, for each gene searched, for each species.
(XLSX)

S1 Text Gene searches. The text file with the DNA sequences used in gene searches, with GenBank accession numbers included.
(TXT)
