# Supplementary material for: Phylogenomics and Divergence Dating of Fungus-Farming Ants (Hymenoptera: Formicidae) of the Genera Sericomyrmex and Apterostigma
Source: PLoS One. 2016 Jul 28;11(7):e0151059. doi: 10.1371/journal.pone.0151059 (PMC4965065; doi:10.1371/journal.pone.0151059)
Supplement: S1 Fig — Ortholog Hit Ratio values for each of the species sequenced. (PDF) [file pone.0151059.s001.pdf]

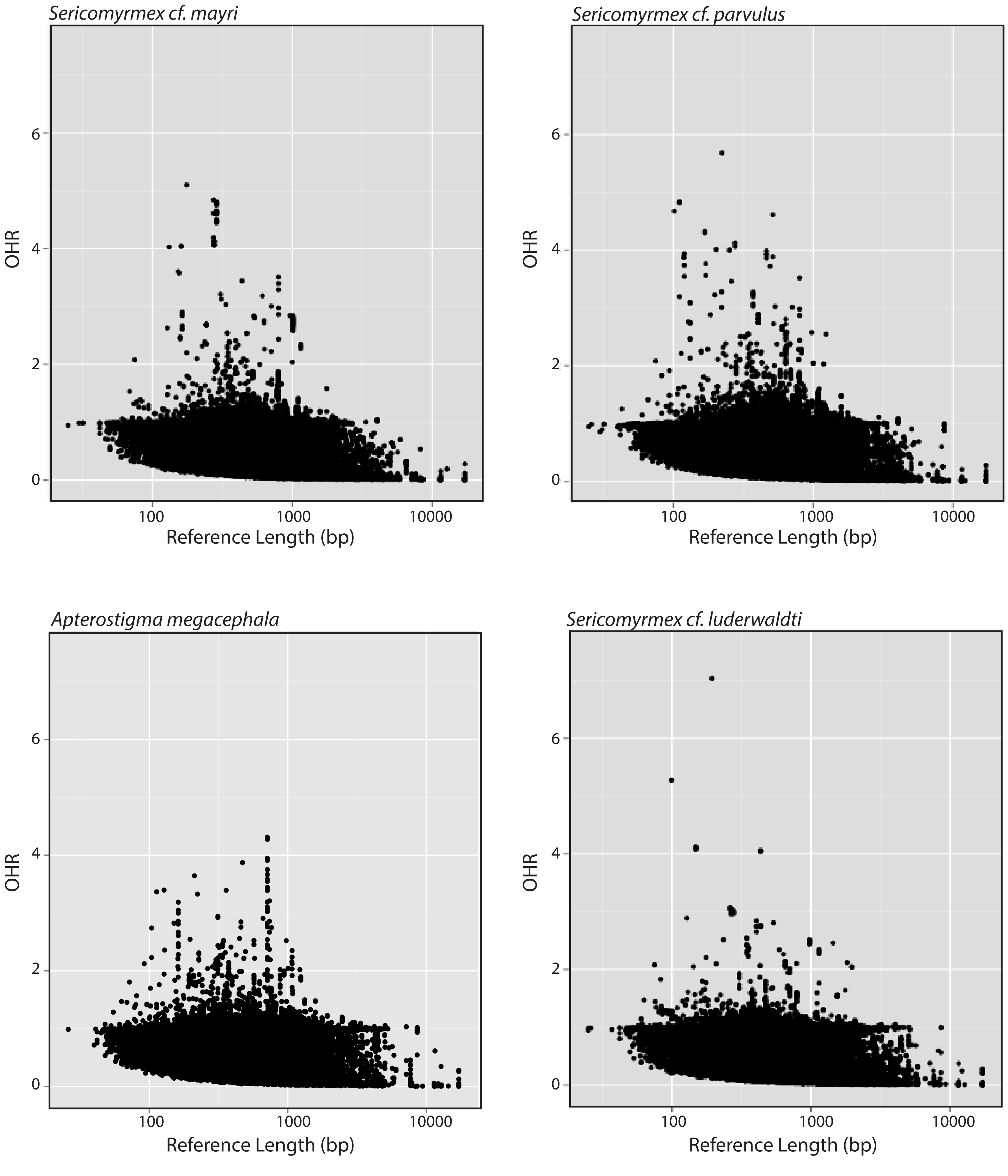

Sericomyrmex cf. mayri
Sericomyrmex cf. parvulus
Apterostigma megacephala
Sericomyrmex cf. luderwaldti
OHR
0
2
4
6
100
1000
10000
Reference Length (bp)
